# Identification of a urine metabolite constellation characteristic for kidney allograft rejection

**DOI:** 10.1007/s11306-018-1419-8

**Published:** 2018-08-30

**Authors:** Miriam Banas, Sindy Neumann, Johannes Eiglsperger, Eric Schiffer, Franz Josef Putz, Simone Reichelt-Wurm, Bernhard Karl Krämer, Philipp Pagel, Bernhard Banas

**Affiliations:** 1 Department of Nephrology, University Hospital Regensburg, Regensburg, Germany; 2 numares AG, Regensburg, Germany; 3 Fifth Department of Medicine, University Medical Center Mannheim, Mannheim, Germany

**Keywords:** Metabolomics, NMR-spectroscopy, Kidney rejection, Diagnostic model

## Abstract

**Introduction:**

Allograft rejection is still an important complication after kidney transplantation. Currently, monitoring of these patients mostly relies on the measurement of serum creatinine and clinical evaluation. The gold standard for diagnosing allograft rejection, i.e. performing a renal biopsy is invasive and expensive. So far no adequate biomarkers are available for routine use.

**Objectives:**

We aimed to develop a urine metabolite constellation that is characteristic for acute renal allograft rejection.

**Methods:**

NMR-Spectroscopy was applied to a training cohort of transplant recipients with and without acute rejection.

**Results:**

We obtained a metabolite constellation of four metabolites that shows promising performance to detect renal allograft rejection in the cohorts used (AUC of 0.72 and 0.74, respectively).

**Conclusion:**

A metabolite constellation was defined with the potential for further development of an in-vitro diagnostic test that can support physicians in their clinical assessment of a kidney transplant patient.

**Electronic supplementary material:**

The online version of this article (10.1007/s11306-018-1419-8) contains supplementary material, which is available to authorized users.

## Introduction

According to the Health Resources & Services Administration, 19,061 patients have received a kidney in 2016 in the USA (Procurement and Network 2017). Despite great improvements in immunosuppressive treatment regimens, allograft rejection is still a substantial threat and one of the biggest concerns faced by both patients and their physicians.

Optimal therapeutic response to a rejection episode depends on reliable diagnostics in order to detect the condition quickly. But even today, many nephrologists find the available diagnostic tool kit lacking. Standard parameters like serum creatinine indicate an impaired glomerular filtration rate but are not specific for a rejection. Histopathological evaluation of ultrasound guided biopsies of the transplanted kidney represents the current gold standard for diagnosing a rejection. However, the procedure is not without risk—hematoma, gross hematuria, hydronephrosis or even graft loss are rare but real complications (Preda et al. 2003; Tsai et al. 2016; Wilczek 1990). Furthermore, the method depends on the availability of an experienced pathologist and relies on the assumption that the tissue core is representative of the entire organ.

Therefore, great efforts have been made to find new biomarkers that complement the current diagnostic toolkit or may even replace graft biopsy in the future (Bassi et al. 2017; Bell et al. 1991; Foxall et al. 1993; Le Moyec et al. 1993).

From a practical point of view, appropriate single biomarkers would certainly be helpful in clinical routine. Unfortunately, it is quite likely that for many diseases and conditions there is no single “silver bullet” biomarker elucidating the complex underlying pathophysiological processes. However, physicians have always integrated multiple sources of diagnostic information in their daily work: patient history, findings from physical examinations, laboratory parameters and imaging techniques are used in conjunction in order to find the correct diagnosis. The same principle can be transferred into the realm of laboratory diagnostics to some extent: By quantifying a small panel of relevant metabolic markers and evaluating them in a quantitative model that assesses the likelihood of a certain condition allows the creation of a characteristic metabolite constellation. Modern tools from the realms of artificial intelligence and machine learning can help to identify important metabolites that are then combined in a final, ideally simple and robust, model. The modelling step is important because drawing correct conclusions from such panels becomes complicated very quickly as the number of variables increases and the relation between them becomes more complex. As a result, the physician is not represented with the raw values of such a metabolite constellation but a *metabolite rejection score* that corresponds to the probability of a condition or its severity, depending on the model in question.

In this work, we describe our quest for a novel diagnostic tool suited for supporting the physician in monitoring kidney transplant patients by building a metabolic marker constellation from patient samples that is indicative of organ malfunction—graft rejection in particular. While most laboratory diagnostics is carried out in blood samples, it appears reasonable to use urine in the particular case of the kidney because, a priori, it seems plausible that physiological changes in the kidney should have an immediate effect on the production and composition of urine.

As our analytical platform, we chose NMR-spectroscopy which has been successfully used as a research tool for decades. The method is capable of detecting hundreds to thousands of organic compounds in a single measurement without complicated pre-analytical manipulations making it ideal for a metabolomic approach to in-vitro diagnostics.

## Materials and methods

### Cohorts

Our overall strategy for building a diagnostic test based on metabolic constellations is to work with three independent sample cohorts: one for training the model, a second for testing and a limited number of final tuning steps and, finally, a validation cohort. In this work, we describe the work of training and establishing the model while the clinical validation using a prospective cohort will be described separately.

For training and testing, we used two separate cohorts, both of which were collected at the transplant center of the University Hospital Regensburg, Germany. The work is covered by the ethics approvals 04/056 and 03/082 (both University of Regensburg, Germany) by the responsible IRB at the University.

Our training cohort consisted of 1883 urine samples taken from 180 renal transplant patients at the University Hospital Regensburg, Germany. The samples were collected between 2008 and 2010 and had been biobanked for future use. In this work, we analyzed them retrospectively. In addition to the samples, we received basic patient characteristics like sex and age as well as the date of transplantation and, of course, the histopathological findings of the graft biopsies.

For the test cohort, 589 samples were prospectively collected between 2015 and 2016 at the same center by taking urine samples from 178 patients visiting the kidney transplant aftercare clinics for routine follow-up or because of specific health problems. Therefore, the samples are independent in the sense that they have been collected over a completely different time period. The only clinical data we collected for this set was the date and result of the pathology reports on kidney graft biopsies performed at or shortly after the visit on which a urine sample was taken.

Hematuria is a frequent observation early after transplantation and can interfere with our metabolic analysis. Therefore, we only included in this work only samples that were taken at least 14 days post transplantation and leave it to be determined later, how early such a test can be used.

### Case/control definition

All transplant biopsies were evaluated by both an experienced nephropathologist and a nephrologist and classified according to the BANFF97 schema (Racusen et al. 1999). Regarding the training cohort, a biopsy result was labeled *case* in case of an acute cellular rejection (BANFF 4, all grades) and *control* if no rejection was found (BANFF 1). Antibody mediated rejections (humoral rejection, BANFF 2), borderline changes (BANFF 3) and chronic rejections (BANFF 5) were excluded from the analysis unless found in conjunction with an acute cellular rejection. Urine samples were not necessarily all taken on the day of a graft-biopsy. All patient samples taken in a time window of up to 7 days before the biopsy (including the day of biopsy) were used in our analysis and labeled *case* or *control* according to the outcome of the corresponding biopsy. In addition, we also labeled all samples from patients who were never subjected to biopsy or whose biopsies were all negative as *control*.

In case of the test cohort, all samples associated with a confirmed positive or negative biopsy were labeled *case* or *control*, respectively (further referred to as *strict* setting). Furthermore, we also considered a variation of this definition for the analysis of the test cohort (further referred to as *extended* setting). Here, we additionally included samples that were not supported by a biopsy as *control* samples.

### Sample handling and preparation

Mid-stream spot urine samples were collected in standard plastic urine cups and aliquots of 1.8 ml were transferred into 2.0 ml sample tubes. The aliquots were frozen at − 20 °C within a few hours after collection. For NMR measurements, aliquots were allowed to thaw at room temperature. Upon complete thawing, 600 µl of the urine sample were mixed with 150 µl of *Axinon urine additive solution* (numares AG, Regensburg, Germany) in a centrifuge tube. The samples were centrifuged @ 20,000×*g* for 10 min at 20 °C and 600 µl of the supernatant was transferred to 5 mm NMR tubes and kept at 2–6 °C until measurement.

### NMR measurements

All measurements were carried out on a Bruker Avance II + 600 MHz NMR spectrometer using a PATXI 1H/D-13C/15N Z-GRD probe. Samples were kept at 2–6 °C in the automated sample changer (SampleJet) and brought to the target temperature of 37 °C in the integrated pre-heating block before measurement. We used a standard pulse program with 30° excitation pulse and pre-saturation for water suppression (zgpr30).

Samples with sufficient volume passing a visual inspection were measured in batches of up to 93 samples per rack. In addition to the patient samples, each rack included one *Axinon urine calibrator* sample and two *Axinon urine control* (numares AG, Regensburg, Germany) samples (positioned at the beginning and the end of a rack) in order to assure ideal measurement and reproducibility conditions throughout the run.

### Signal analysis

NMR spectra were referencing to TSP (trimethylsilylpropanoic acid) to ensure comparability on the ppm-axis. They underwent automatic phase correction and baseline correction before further analysis. Subsequently, we applied automatic standardization and calibration procedure to minimize between-device, between-day and between-run effects. Finally, we applied a quality control filter based on spectral properties, such as offset and slope of the baseline in selected spectral regions as well as properties of selected signals, e.g. signal position, shape and width.

We applied the instruments water suppression program, and excluded the region containing the residual water signal (4.5–5.0 ppm) from further analysis.

### Data processing and modelling

All statistical analyses were carried out with the R statistical software v3.0.2 (R Core Team 2014). Modelling and feature selection was carried out with package mlr v2.3 (Bischl et al. 2016) for random forest models (using the integrated randomForest package v4.6-7) and the function glm (generalized linear models) from stats for logistic regression. ROC (receiver operating characteristic) curves and AUC (area under the curve) computations were carried out using the package pROC v1.5.4.

Signal fitting was carried out in C# using the library NMath v5.2.0.3 (Centerspace Software, Corvallis, Oregon, USA) for numerical work.

## Results

### Strategy outline

**Fig. 1:**
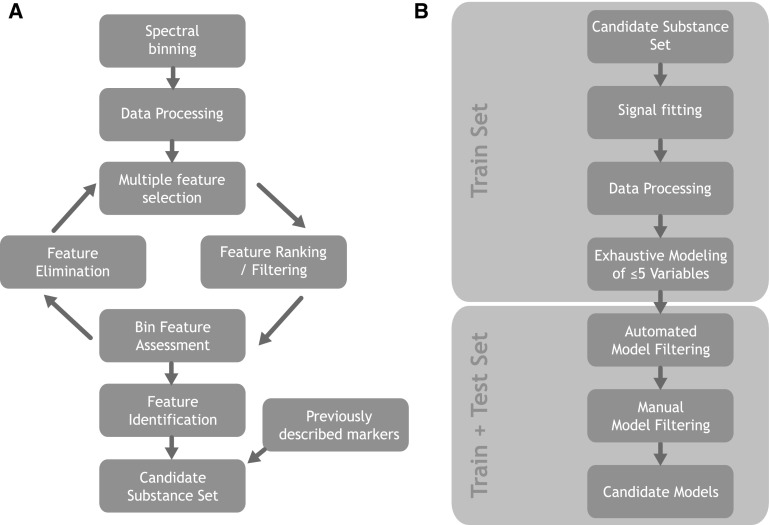
Overview of our modelling strategy. **a** Process from binned spectra towards candidate substance set. **b** Modelling with fitted metabolites towards final candidate models

In this work, we carried out two distinct phases as illustrated in Fig. 1. In the first phase, we used the popular spectral binning approach. The goal of this phase was to discover a set of candidate bin-features that seem to contribute to classification, lend themselves to proper quantification and can be positively identified. In the second phase, we switched to fitting the signals of interest in the candidate bins for robust quantification and re-built the classification model from scratch based on the properly quantified metabolites. Finally, we went through the process of choosing our final classification model by integrating statistical results with physiological considerations.

The first phase (Fig. 1a) started by binning the NMR spectra followed by data processing steps such as applying transformations, centering and scaling. After that we went through several iterations of statistical feature selection (“multiple feature selection”), feature filtering and ranking, visual inspection/assessment of candidate bins and elimination of features that did not pass our criteria. Afterwards the preliminary bin-feature set was subjected to thorough metabolite identification efforts. In addition to the remaining, positively identified, features we added metabolites to the set that had been previously described as candidate markers for kidney rejection or related kidney problems. The resulting final substance set was allowed to enter phase two.

The second phase (Fig. 1b) started by applying signal fitting to the peaks of interest. The results underwent transformation, centering and scaling and were used as the input for the subsequent exhaustive modelling step. At this point the test cohort was used for the first time: in addition to assessing the classification performance, independently, it helped in guiding the very last steps in picking the final model destined to be implemented in an in-vitro diagnostic product.

### Data flow

As mentioned in Sect. 2, we subjected every NMR spectrum to a qualification step designed to reject spectra that are of inferior quality.

**Fig. 2:**
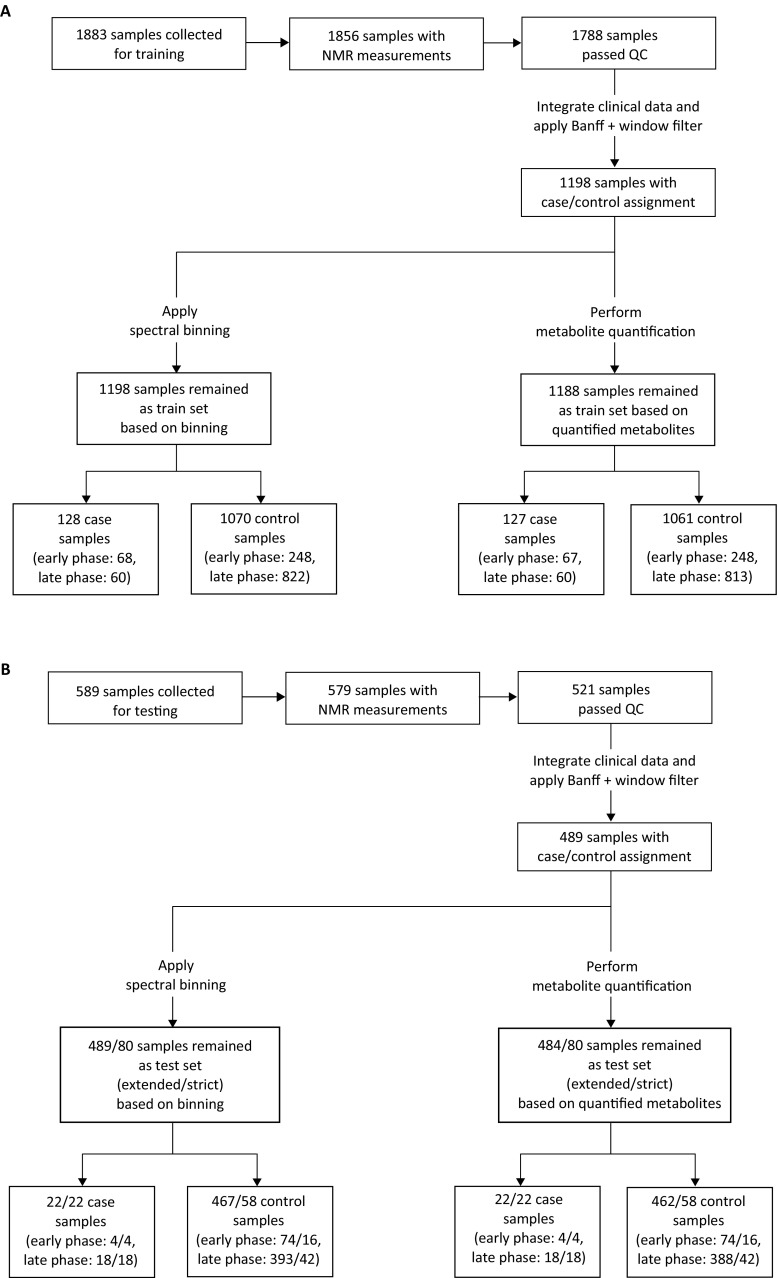
Data flow of analyzed samples in the training (**a**) and test cohort (**b**). Urine samples with sufficient volume for NMR measurement that passed a visual inspection were measured and the spectra were subjected to automatic quality control. In order to classify the remaining valid spectra as either *case* or *control*, they were integrated with the clinical data. In case of the training cohort, samples were filtered according to the biopsy result and the distance between the sample collection and the biopsy. All classified samples were then used for statistical modelling in two phases: first based on binned spectra and then based on quantified metabolites. At this point, two subsets were distinguished: samples from the early phase (i.e. day < 15 after transplantation) and late phase (i.e. day ≥ 15 after transplantation)

The training cohort initially comprised 1883 collected urine samples (see Fig. 2a). 1788 of the sample spectra successfully passed the qualification criteria and were integrated with the clinical data. After applying the windows for defining case and control samples we were left with 1198 classified samples (128 cases and 1070 controls). 882 (60 cases and 822 controls) of those samples originated from the late phase (day ≥ 15 after transplantation) and were used in the two following modelling phases (bin-based and metabolite-based). Please note that depending on the combination of metabolites used to build a model, less data may be available as the underlying metabolite quantification (fitting) may fail to return a valid value.

The test cohort consisted of 589 original urine samples yielding 521 spectra after quality control and 489 classified samples (see Fig. 2b). In the strict setting, 60 samples (18 cases and 42 controls) were left in the late phase, while the extended setting yielded 411 samples (18 cases and 393 controls).

In the following sections, all steps shortly described in the above strategy outline are explained in more detail.

### Modelling of spectral data

NMR spectra were segmented into bins of equal length yielding 377 bins. In order to account for the huge differences in urine concentration, the bins were normalized by the total integral of the respective spectrum. Furthermore, the bins (hereafter referred to as features) were further processed (cubic-root transformation and autoscaling) for the subsequent statistical analyses (for detailed information see section’ Spectral binning & data processing’ in the Supplementary Material).

In order to select only those features that are most useful for our modelling task, we applied feature selection using *random forest* (RF) models. This is a necessary step as many spectral features are uninformative or redundant. In this work, an iterative approach was used based on automatic feature selection and manual elimination of bins by careful visual assessment. This procedure was carried out until all selected bins were deemed acceptable (for detailed information see sections ‘Multiple Feature Selection’ and ‘Bin Feature Assessment and Feature Elimination’ in the Supplementary Material).

### Candidate marker set and quantification

In order to move forward from a black-box situation to a set of known metabolite markers, we next aimed at identification of the signals in the bins. Initial hypotheses were drawn from comparisons to the human metabolome database (HMDB) (Wishart et al. 2013) and proprietary reference spectra at numares. The initial candidates were evaluated for plausibility of being present in human urine and eventually confirmed or ruled out by spike-in experiments. This process was carried out until a substance assignment was possible with confidence. Finally, our candidate substance set comprised 8 distinct metabolite signals.

Of course, the downsides of spectral binning may result in missing some valid markers during feature selection, e.g. when interference or substantial background mask the contribution of the metabolite resulting in a low classification contribution. Therefore, we carried out a thorough literature search for potential markers identified in the context of kidney allograft rejection or other kidney problems. In the literature, there are reports that associate dimethylamine (DMA) and urea with kidney graft rejection in NMR based studies (Bell et al. 1991; Foxall et al. 1993; Le Moyec et al. 1993) both of which did not show up as significant in our own analysis above and were judged to be of reasonable bin quality. Accordingly, our set of candidate markers now comprised the following metabolites: Alanine, Citrate, Dimethylamine (DMA), Glucose, Glucuronate, Hippurate, Lactate, Phenylacetylglutamine (PAQ), Trigonelline and Urea. In addition to these candidate markers, we also quantified creatinine which is required for normalization in urine.

For robust quantification we fitted pseudo-voigt functions to all candidate signals described above. This method allows to also determine goodness of fit and thus detect, when the quantification is unreliable or even impossible in a specific sample—e.g. due to interference by other metabolites. This is of particular importance for this work because we are aiming at developing a test for real world diagnostics.

### Data processing

Before statistical modelling, the intensity distribution of metabolite peaks quantified by peak-fitting were explored by visual inspection (histograms) and several different transformations (log, log-modulus, arctan, cubic-root) were applied. We obtained the best results with respect to symmetrical distribution with log and cubic-root transformations. In contrast to spectral bins, the metabolite quantification method is not plagued by occasional negative values. Accordingly, we settled on the canonical log transformation. Normalization to creatinine values was used to compensate for urine concentration and centering and scaling was used before moving forward.

### Exhaustive modelling on fitted signals

At this point, a new iteration of feature selection is necessary in order to find the final set of markers to include in the classification model. In contrast to the initial situation we now do not face a high-dimensional classification problem, because the feature space was reduced from 377 (bins) to 10 quantified signals. This allows for an exhaustive selection strategy in which we evaluated all $$\mathop \sum \limits_{{i=1}}^{5} \left( {\begin{array}{*{20}{c}} {10} \\ i \end{array}} \right)=$$ 637 models of up to 5 metabolites drawn from the 10 final metabolite candidates.

In the presence of only 10 independent variables a machine learning model such as a random forest model is probably overkill. Therefore, we decided to also fit a multiple logistic regression at this stage and compared the performance. It turned out that the random forest did not outperform the logistic regression model under these conditions. As the latter is much simpler, easier to explain and understand intuitively, we followed the principle of parsimony and picked the logistic regression model over the random forest.

### Automated model filtering

Mathematically, it is easy to simply pick the best performing model (e.g. by cross-validated AUC), but in many situations, the performance estimate for the second, third or kth best performing models is not that far off.

In order to reach a decision of the final model suitable for our purpose we considered both statistical and biological aspects. Upfront, we disregarded all models that did not achieve an AUC of ≥ 0.75 in the ROC analysis in the late phase training cohort. This step left us with 424 candidate models with AUC values ranging from 0.75 to 0.86 in the late phase training cohort.

At this point, it is hard to pick a single model solely based on performance, because we do not have the thousands of samples that would be required to narrow the confidence interval of performance measures to the point that would allow a clear cut decision. Therefore, we decide to open the test set and assess the performance of the candidate models in these independent samples.

Models that failed to perform with an AUC of ≥ 0.70 in the new samples (late phase only) of the two test sets were dropped. By doing so, 33 models were left for the strict and 78 models for the extended setting of the test cohort.

### Manual model filtering

In an initial assessment of the metabolites, we investigated trigonelline as a potential biomarker for kidney rejection. Trigonelline is a natural component in green coffee beans indicating a correlation between the urinary trigonelline and the dietary intake of coffee. This implies that trigonelline may be useful as a biomarker for coffee consumption (Lang et al. 2011) even though small amounts of the molecule may also be produced by gut bacteria. To avoid any potential disturbances of the test system caused by coffee intake it was finally decided to exclude all models that used trigonelline as a feature, leaving a total of 33 and 60 candidates for the strict and extended setting, respectively.

**Fig. 3:**
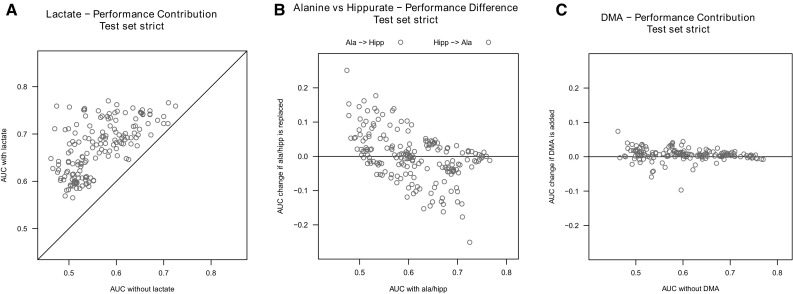
Manual model selection based on AUC comparison in the test set. All graphs show the effect on AUC of different variations of models. **a** Analysis of effect of adding Lactate. Models substantially improves in performance when lactate was added as another independent variable—all data points are found above the diagonal. **b** Analysis of effect of exchanging Alanine and Hippurate: on average, AUC was greater, when Alanine was used as an independent variable instead of hippurate—the magenta points are mostly found above the blue points, although the absolute improvement is small. **c** Analysis of effect of adding DMA. Adding DMA to a model seems to improve performance (data pints above the black line). However the amount of performance gain is negligible at 0.01–0.04 points in the AUC

Next, we examined the suitability of lactate as an element of our model. To begin with, lactate has been described in the literature in the context of kidney rejection (see Sect. 4.1 below) adding to our own evidence. We also compared all non-trigonelline models containing lactate to their counterparts without lactate—i.e. models that differed in the set of features only in the presence or absence of lactate and found that on average models containing lactate as a feature performed appreciably better than without (see Fig. 3a; median absolute difference between the AUC of the models with lactate and their counterparts without lactate was 0.096 for the strict setting). Thus, we excluded models not containing lactate yielding 33 and 54 candidates for the strict and extended setting, respectively.

During inspection of the remaining models, we observed that the well performing models either contained alanine or hippurate but almost never both which is not very surprising, because these two metabolites are significantly correlated in our data. As the alanine containing models tended to perform slightly better than those containing hippurate (see Fig. 3b; median absolute difference between the AUC of the models was 0.035 for the strict setting), we picked alanine over hippurate at this step. By doing so, 11 models were left for the strict and 17 models for the extended setting in the test cohort.

Finally, we examined DMA and found the performance contribution to be of little practical value (see Fig. 3c): the median absolute difference between the AUC of the models containing DMA and their counterparts w/o the metabolite was 0.008. Therefore, it was decided not to include DMA. After this last manual filtering step, we were left with 7 and 11 models for the strict and extended setting. All seven models from the strict setting were included in the list for the extended setting. Thus, we decided to use the intersection of the two lists for further analyses.

### Selecting the final model

**Fig. 4:**
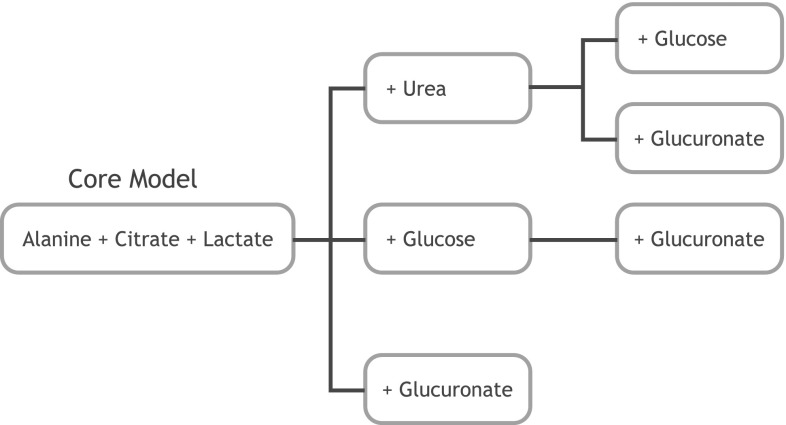
Final candidate models. After selecting by model performance and assessing some feature alternatives (e.g. alanine vs. hippurate) we were left with a core model that comprises the features alanine, citrate and lactate. In addition there are a few models that add urea, glucose and/or glucuronate to that core feature set

At this point, our candidate model set was narrowed down to seven models that represent variations on a core feature set (alanine, citrate, lactate). Figure 4 depicts the relation between these final model candidates. All of these seven models show very similar performance so the last selection steps were purely based on biological and technical considerations.

While glucose should not be present in appreciable concentrations in the urine of healthy people, glucosuria is not that uncommon in the cohort of interest. That means that a test using glucose as a feature is prone to interference by glucosuria and thus undesirable. Therefore, we decided to drop all models containing glucose as a variable.

Glucuronate does not have that problem directly, but it’s signal is located in the immediate neighborhood of the glucose signal requiring a simultaneous quantification of both metabolites in order to avoid interference between them. Furthermore, glucuronidation is a well known mechanism for renal elimination of xenobiotic substances from the body. A xenobiotic is a chemical compound that is not endogenously produced within an organism (such as drugs) and is therefore a foreign substance that can reach toxic concentrations. Glucuronidation is a major pathway of xenobiotic biotransformation where the drug in question is conjugated with glucuronate in order to increase their water solubility and thus allow them to be filtered or excreted by the kidney. In the NMR spectrum we cannot reliably distinguish free glucuronate from the glucuronides. On one hand, a molecule that is part of a renal elimination mechanism may be a valid indicator of a kidney impairment, on the other hand it is possible that we are seeing a confounding effect related to the drug load of the patient which could be related to rejection. Therefore, we decided not to keep this metabolite in the final set.

The situation for urea is different, because the molecule is a well known marker for kidney function, at least in serum, so it seems much more reasonable to include it in the model on physiological grounds (see below for detailed discussion in Sect. 4).

As a result of the statistical and biochemical/physiological considerations explained above, we chose alanine, citrate, lactate, urea and creatinine (for normalization) as our final feature set resulting in the following logistic regression model:

$$Score=100 \times \frac{1}{{1+{e^{ - \omega }}}}$$

where ω = − 3.0048615 − 0.2527461 × *I*_*alanine*_ − 0.8224731 × *I*_*citrate*_ + 0.9502339 × *I*_*lactate*_ + 0.2529190 × *I*_*urea*_ and *I*_*x*_ is the creatinine normalized, log-transformed, centered and scaled signal intensity of metabolite *x* in the sample.

### Performance

**Fig. 5:**
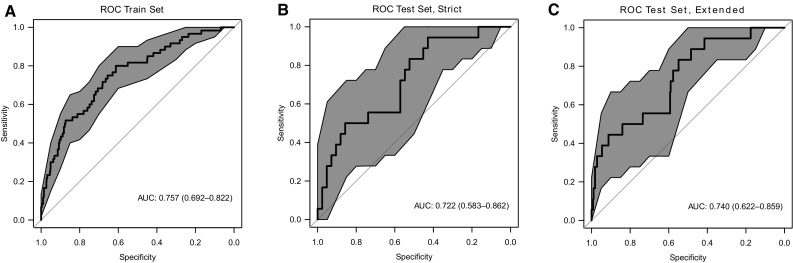
Performance in training and test cohort. ROC curve of fitter model for late phase. The blue area indicates 95% confidence intervals of the ROC curves. **a** Performance in the training set. **b** Performance in the test set using the strict case/control definition based on biopsy results. **c** Performance in the test set using the extended case/control definition that additionally includes control samples when no biopsy was performed due to lack of clinical symptoms

In order to estimate the expected classification performance, we generated receiver operator characteristic (ROC) curves and computed the area under the curve as a measure of performance for both the training and the test cohorts. For the training set, we achieved an AUC value of 0.76, and for the test set the observed values were 0.72 and 0.74 for the strict and extended setting (Fig. 5). Of course, it is problematic to trust training set performance, because the danger of overfitting cannot be neglected, even if proper procedures are followed. Nevertheless, training set performance is an important indicator of the upper bound of performance that can be expected in independent data.

## Discussion

### Physiological assessment of markers

While statistical performance is important, patho-physiological plausibility of a given model must be addressed in order to detect possible artifacts and generate an overall understanding of the predictor as a whole. Therefore, we evaluated all metabolites that were chosen in one of the competing top performing models with respect to their biochemical and pathophysiological roles. Of course, it is not a reasonable requirement that all markers used in the model have been described in the context of kidney graft rejection, previously, but we aimed for plausible roles in the rejection process or kidney function itself.

Our urinary metabolic constellation attempts to address the complexity and multifactorial aspect of organ transplant complications. The individual biomarkers might represent the downstream products of cellular activity, lymphocyte invasion and the immediate molecular response to stresses associated with rejection episodes. The most relevant resonances for evaluating renal function after transplantation in our hands were those of citrate, lactate, alanine, and urea normalized to creatinine excretion.

Urinary citrate excretion is a common tool in the differential diagnosis of renal tubular acidosis (Buckalew 1989). The key regulatory enzymes directly associated with citrate production are mitochondrial aspartate aminotransferase, pyruvate dehydrogenase, and mitochondrial aconitase. Low urinary citrate might therefore be an indicator of renal tubular acidosis, given our observation of a strong negative correlation with transplant rejection. In line with this conclusion, Le Moyec et al. demonstrated that citrate is a urinary marker that can identify transplanted kidneys with a favorable prognosis (Le Moyec et al. 1993). They analyzed urine from 39 patients who underwent renal transplantation by NMR spectroscopy. These findings were also supported in animal models, describing dose-related decreases in citrate excretion as indicator of mercury-induced nephrotoxicity in rats (Nicholson et al. 1985). Urinary citrate was also validated to be indicative for chronic kidney disease in general (Posada-Ayala et al. 2014).

In our cohorts, urinary lactate showed a strong positive correlation with rejection episodes. A shift towards anaerobic metabolism might indicate respiratory chain dysfunction in mitochondria of the kidney during rejection and a subsequent accumulation of lactate. Similar findings were reported by Thirumurugan et al. who compared urinary lactate levels in pediatric patients suffering from Fanconi syndrome with those of healthy children and with those with other renal diseases (Thirumurugan et al. 2004). Urinary lactate was increased in Fanconi syndrome. The authors suggest that the increase might be associated with reduced lactate reabsorption in the proximal tubule. Hence, urinary lactate/creatinine might reflect disordered proximal renal tubular function during cellular transplant rejection.

MacPherson et al. described that tubulitis during acute rejection induced altered aminoaciduria shortly before clinical manifestations of acute rejection became evident (MacPherson et al. 1991). These alterations in urinary amino acid excretion occurred several days before changes in urinary protein excretion or the serum concentrations of urea and creatinine. Hence, the deteriorated alanine/creatinine ratio observed in our study might at least in part reflect tubular cell disturbances caused by acute tubulitis.

Blood urea nitrogen is a well-accepted indication of renal health. The liver produces urea in the urea cycle as a waste product of protein digestion. The main pathological cause of an increase in serum urea is a decrease in glomerular filtration rate, suggesting renal failure. The urinary urea concentration positively associated with kidney transplant rejection in our study might be reflective of increases in the respective serum levels. These were described to be predictive for kidney transplant failure (Moore et al. 2011). The authors report on the development and validation of a composite risk score to predict kidney transplant failure. Serum urea in conjunction with eGFR, recipient age, race, albumin levels, and prior acute rejection significantly predicted overall transplant failure.

In summary, our urinary markers indicate kidney transplant rejection, because they might reflect tubular dysfunction on the mitochondrial level, which means a disturbed energy metabolism due to hypo-perfusion, organ swelling, and respiratory chain dysfunction. This might be accompanied by renal tubular acidosis, deteriorated co-transport in the proximal tubule and decreased glomerular filtration. In contrast, we could not detect any indication that soluble lymphocytic metabolites are entering the urine in significant amounts during rejection. The biochemical mechanisms of invading lymphocytes on tubular function during rejection remain largely unclear.

### Outlook

In this work, we have presented our modelling strategy and its results in the training set as well as a test cohort. The latter was not used in the training process. However, we did use it in selecting the final model. Therefore, the next obligatory step is the performance assessment of our metabolite rejection score in a fully independent, prospective clinical trial. The results of that study will be published separately.

## Electronic supplementary material

Below is the link to the electronic supplementary material.

Supplementary material 1 (DOCX 21 KB)
