# Supplementary material for: Identification of a urine metabolite constellation characteristic for kidney allograft rejection
Source: Metabolomics. 2018 Aug 30;14(9):116. doi: 10.1007/s11306-018-1419-8 (PMC6133122; doi:10.1007/s11306-018-1419-8)
Supplement: Supplementary file 1 — Supplementary material 1 (DOCX 21 KB) [file 11306_2018_1419_MOESM1_ESM.docx]

Supplementary Material to
Identification of a urine metabolite constellation characteristic for kidney allograft rejection

Miriam Banas, Sindy Neumann, Johannes Eiglsperger, Eric Schiffer, Franz Josef Putz, Simone Reichelt-Wurm, Bernhard Karl Krämer, Philipp Pagel and Bernhard Banas

Miriam Banas, Department of Nephrology, University Hospital Regensburg

E-mail: miriam.banas@klinik.uni-regensburg.de

Sindy Neumann, numares AG, Regensburg

E-mail: sindy.neumann@numares.com

Johannes Eiglsperger, numares AG, Regensburg

E-mail: johannes.eiglsperger@numares.com

Eric Schiffer, numares AG, Regensburg

E-mail: eric.schiffer@numares.com

Franz Josef Putz, Department of Nephrology, University Hospital Regensburg

E-mail: franz-josef.putz@ukr.de

Simone Reichelt-Wurm, Department of Nephrology, University Hospital Regensburg

E-mail: simone.reichelt-wurm@ukr.de

Berhard Karl Krämer, Fifth Department of Medicine, University Medical Center Mannheim

E-mail: bernhard.kraemer@medma.uni-heidelberg.de

Philipp Pagel, numares AG, Regensburg

E-mail: philipp.pagel@numares.com

Bernhard Banas, Department of Nephrology, University Hospital Regensburg

E-mail: bernhard.banas@klinik.uni-regensburg.de

# Material and Methods

# Spectral binning & data processing

Spectra were divided into 403 bins of 0.04 ppm width positioned with 50% overlap between consecutive bins, thus covering the range from 0.96 to 9.04 ppm. The size of our spectral bins was chosen to allow a typical peak to reasonably fit into a bin while not expanding the bin to an extent that would make it highly likely that multiple signals are included in one bin. Overlapping bins allow the subsequent feature selection to pick bins that best accommodate the peaks of interest in cases where non-overlapping bins would not frame a peak very well. However, the presence of more than one signal is by no means impossible, so further efforts must be made, later, to end up with clean quantifications of each marker (candidate). After excluding the water signal, a total of 377 bins remained.

Depending on water intake and kidney function, urine concentration varies widely between samples. Accordingly, urine analytics is either done on urine collected over large time intervals (typically 24h) or standardized to the creatinine concentration (i.e. dividing all concentrations relative to the creatinine concentration). To address this concentration issue, we applied two different methods in the early (i.e. bin-based analysis) and late (i.e. signal fitter based analysis) phases our work. Initially, we decided against using a bin containing a creatinine signal and used the total integral of the spectrum for normalization. Once we had established signal fits for our metabolites of interest, we also used a creatinine signal fitting approach for normalization.

Before further analysis, the intensity distribution of all bins was assessed by visual inspection. In order to correct the pronounced skewness observed in most of the bin intensity distributions, we tested several common transformations on the data (log, log-modulus, arctan, cubic-root). Both the log and cubic-root transformation resulted in a substantial improvement with respect to distribution symmetry (data not shown). Because binned data can yield negative values under certain conditions, we chose the cubic-root transformation over the log. Centering and scaling was used to normalize the intensity of all bins before statistical analysis.

# Multiple Feature Selection

At this stage, the primary goal was to identify a reasonably small set of candidate features (bins) for the development of a multi-marker model which will then need to be refined. No effort was made, at this point, to identify all features as this is done at a later stage of our work.

The problem at hand is of high dimensionality, thus it makes sense to use a modelling algorithm that is well suited for this type of problem. We chose *random forest* (RF) models for our work because this type of model is well established as a robust method for machine learning problems in a large number of different situations and reliable implementations are available (e.g. (Liaw and Wiener 2002)). Furthermore, random forests are reputed to be fairly robust against overfitting and imbalanced data with respect to cases and controls ((Breiman 2001)).

Feature selection is an important step in any *omics classification approach. The goal is to reduce the initially large number of features to a much smaller set that is then used to fit the actual model. This is particularly important in metabolomics as the features are highly redundant such that multiple spectral signals can correspond to the same metabolite. Furthermore, some regions in a spectrum are uninformative as they do not contain any signals and correspond to noise. Generally feature selection is very important, because models with a large number of independent variables (features) are prone to overfitting. I.e. they will yield near perfect classification results in the training set by simply using random differences between classes that happen to be present in the training data. Once applied to new data, performance collapses because the differences used were not actual markers for the groups.

In this work, an iterative approach was used based on automatic feature selection and manual elimination of bins by careful visual inspection. For the former, a wrapper approach ((Kohavi and John 1997)) was applied based on random forest as the underlying learning technique and sequential floating forward search ((Pudil, Novovicova, and Kittler 1994)). This search strategy starts with an empty model and, with each further step, chooses the best model out of all models with one more and one less feature. We restricted the maximal number of features to 10. For a more robust feature selection, we rerun the feature selection 50 times with different seeds. Then, we analyzed how often a feature had been among those selected by the method and only considered those bins that had prevailed in at least 10% of the feature selection iterations. Features were ranked by their frequency and then subjected to bin assessment as described below. Thereby, single bins were excluded and a new run of multiple feature selection was started, potentially selecting new bins that required another round of bin assessment. This procedure was carried out in an iterative fashion until all selected bins were deemed acceptable.

# Bin Feature Assessment and Feature Elimination

Not all bins in an NMR spectrum contain meaningful information – some are void of discernible signals, other solely contain broad background signals that are not attributable to individual metabolites but originate from small amounts of protein or blood present in the respective urine sample. By design, feature selection algorithms try to only pick bins that carry real information but it can happen that random differences in baseline correction, phasing or noise-level mislead the selection process. Furthermore, these bins increase the number of features to evaluate and thus contribute to the multiple testing problem without carrying information relevant for classification. Therefore, it is good practice to apply some sort of feature filtering that is not based on the statistical classification problem in order to reduce the feature space before actual modelling.

We subjected all bins selected by the feature selection algorithm to careful visual inspection in the entire cohort. In addition to the problems described above, our NMR experts also excluded bins that did contain signals but were too burdened with signal-interference to allow reliable signal identification, later in the process. In addition, we assessed the positional stability of the candidate peaks and eliminated signals that tended to shift too much for reliable peak calling.

# References

Breiman, L. 2001. “Random forests.” *Machine Learning* 45 (1): 5–32.

Kohavi, Ron, and George H. John. 1997. “Wrappers for Feature Subset Selection.” *Artificial Intelligence* 97 (1): 273–324. doi:[http://dx.doi.org/10.1016/S0004-3702(97)00043-X](https://doi.org/http://dx.doi.org/10.1016/S0004-3702(97)00043-X).

Liaw, Andy, and Matthew Wiener. 2002. “Classification and Regression by RandomForest.” *R News* 2 (3): 18–22. <http://CRAN.R-project.org/doc/Rnews/>.

Pudil, P., J. Novovicova, and J. Kittler. 1994. “Floating search methods in feature selection.” *Pattern Recognition Letters* 15 (11): 1119–25.
